# Ethylene Supplementation Combined with Split Application of Nitrogen and Sulfur Protects Salt-Inhibited Photosynthesis through Optimization of Proline Metabolism and Antioxidant System in Mustard (*Brassica juncea* L.)

**DOI:** 10.3390/plants10071303

**Published:** 2021-06-27

**Authors:** Badar Jahan, Noushina Iqbal, Mehar Fatma, Zebus Sehar, Asim Masood, Adriano Sofo, Ilaria D’Ippolito, Nafees A. Khan

**Affiliations:** 1 Plant Physiology and Biochemistry Laboratory, Department of Botany, Aligarh Muslim University, Aligarh 202002, India; naziabadar.2014@gmail.com (B.J.); meharfatma30@gmail.com (M.F.); seharzebus5779@gmail.com (Z.S.); asim.bot@gmail.com (A.M.); 2 Department of Botany, Jamia Hamdard, New Delhi 110062, India; naushina.iqbal@gmail.com; 3 Department of European and Mediterranean Cultures: Architecture, Environment, Cultural Heritage (DiCEM), University of Basilicata, 75100 Matera, Italy; dippolito.ilaria@libero.it

**Keywords:** antioxidants, *Brassica juncea*, ethylene, nitrogen assimilation, proline metabolism, sulfur assimilation

## Abstract

In the present study, the potential of ethylene as ethephon (an ethylene source) was investigated individually and in combination with split doses of nitrogen (N) and sulfur (S) soil treatments for removal of the damaging effects of salt stress (100 mM NaCl) in mustard (*Brassica juncea* L.). Plants were grown with 50 mg N plus 50 mg S kg^−1^ soil at sowing time and an equivalent dose at 20 days after sowing [N50 + S50]_0d and 20d_. Ethephon at 200 μL L^‒1^ was applied to combined split doses of N and S with or without NaCl. Plants subjected to NaCl showed a decrease in growth and photosynthetic characteristics as well as N and S assimilation, whereas proline metabolism and antioxidants increased. The application of ethephon to plants grown with split N and S doses significantly enhanced photosynthetic efficiency by increasing the assimilation of N and S, improving the concentration of proline and induction of the antioxidant system with or without NaCl. The regulation of ethylene and/or split forms of N and S application may be potential tools for not just overcoming salt stress effects in this species and in related Brassicaceae but also enhancing their photosynthesis and growth potential through increased nutrient assimilation.

## 1. Introduction

A remarkable increase in the population at the global level, combined with speedy industrialization in emergent countries, has caused issues for global food and energy needs. According to the United Nations and Food and Agricultural Organization, the world population will expand to 9.7 billion by 2050, and will face extreme challenges on various fronts, among which attaining food security will be a high-priority issue [1]. However, increasing exposure of plants to abiotic stresses will be a limiting factor in achieving this goal. Among various abiotic stress factors, salt stress is one of the important problems worldwide that limits reliable crop production and food security globally [2]. It causes losses of about USD 27.5 billion and affects an area of approximately 936 Mha yearly nationwide [3,4]. Salt stress induces different physio-biochemical abnormalities, toxic ion uptake as sodium (Na^+^) and chloride (Cl^−^),imbalances of essential nutrients, and dual hyperosmotic effects and disturbs the homeostasis of water, reducing plant growth rates and yield productivity [5,6]. Additionally, salt stress damages cellular membranes through reactive oxygen species (ROS) accumulation. These ROS, which include superoxide anion (O_2_^•−^), hydrogen peroxide (H_2_O_2_), and hydroxyl radical (HO^•^), are toxic, highly reactive [7,8], cause oxidation of proteins and lipids, and may cause DNA damage in different cellular compartments [9]. This results in increased carbonylated proteins and malondialdehyde (MDA) concentrations that are indicators of oxidative stress [10].

To lessen the damaging effects of salt stress, plants have developed several processes that involve antioxidant defense systems, compartmentation of ions, and osmolyte accumulation [5,6]. The enhancement of antioxidant enzymes works as an observable defense strategy under stress that interacts with ROS detoxification [5,8,11]. Presently, for strengthening tolerance mechanisms against salt stress, various master plans are being tested or adopted, including traditional biotechnological and standard methodologies [12]. In the most recent couple of years, efforts have focused on mineral nutrient supplementation and plant growth regulators for improving the development and yield of plants under salt-stress environments [12,13].

Among nutrients, nitrogen (N) and sulfur (S) are involved in several abiotic and biotic stress tolerance mechanisms through the production of osmolytes, plant hormones, and non-enzymatic and enzymatic antioxidants [5,14]. They are essential players in the biosynthesis of essential organic complexes, comprising protein, amino acids, nucleic acids, and several other cellular components [15,16,17]. In addition, S-containing metabolites, including a large range of crucial metabolites such as thiols, reduced glutathione (GSH), amino acids, cysteine (Cys), and methionine (Met), play an important part in the tolerance of salt stress [5,18]. Proline and GSH are important in enhancing salt tolerance as they help in maintaining the osmotic balance and redox state, besides functioning as antioxidants to scavenge ROS. Nitrogen and S are important components of proline and GSH and enhance their concentration. Moreover, the assimilatory pathways of N and S have common linking points, such as cysteine (Cys), and are considered to effect each other [5,19,20] and also ethylene synthesis via Met and S-adenosyl methionine (SAM), a precursor of ethylene (ET). Ethylene has been reported to influence proline concentration to induce salt stress tolerance in *Brassica juncea* [21]. Ethylene signaling has been found to increase the accumulation of proline for osmotic adjustment [22]. It was reported that enhanced activity of ascorbate–glutathione cycle enzymes produced higher concentrations of GSH that reduced salt and excess glucose-induced oxidative stress in *Triticum aestivum* [23]. Salt tolerance was associated with increased relative water content, lower lipid peroxidation, and increased proline and polyamine accumulation accompanied by increased ethylene [24]. The induction of ethylene biosynthesis by GSH has been reported [25].

Ethylene is a stress-responsive gaseous hormone that functions as an important contributor to plant development and growth under abiotic stress conditions [11,26,27]. It has been reported that ET stimulates the stomatal response, permitting further entry of CO_2_ for carboxylation, as well as enhanced photosynthesis [28]. Moreover, ET decreases ROS accumulation induced by high salinity and ultimately enhances plant tolerance to excess salt [29]. Ethylene increases the assimilation of N and controls proline synthesis in plants in both optimal and stress environments [28,30,31,32]. A recent study showed that the supplementation of ET and S regulates ABA concentration and the antioxidant system and enables the responses of stomata, chloroplast ultrastructure, and photosynthetic characteristics in *B. juncea* experiencing salt stress [27]. The involvement of ethylene in salt tolerance through activation of the ethylene-insensitive 3 (EIN3) and EIL1 (EIN3-LIKE 1) transcription factors that mediate *SIED* (salt-induced and EIN3/EIL1-dependent) genes involved in ROS scavenging and acceleration of the pathway for salt tolerance has been shown [33]. During hypoxia, early ethylene entrapment sends signals to increase the stability of ERFVII by decreasing nitric oxide (NO) formation through increased formation of phytoglobin1 (PGB1). PGB1 acts as a NO-scavenger and links ethylene signaling to O_2_ sensing, leading to hypoxia tolerance. Ethylene response in hypoxia to flooding stress has been demonstrated through analysis of the genes responsible for hypoxia tolerance [34]. In the present study also we observed ROS scavenging through ethylene under salt stress.

The importance of split doses of N and S in salinity tolerance was delineated in the study of Jahan et al. [5]. Excess ethylene has been found to restrict nutrient absorption by the root as reported by Pandey et al. [35]. They reported that in compacted soil, ethylene concentration in the root tissues increases and activates cellular signaling cascades that stop root growth and productive nutrition. As discussed earlier, ET shows positive interaction with both N and S, is involved in enhancing the assimilation of both these nutrients, and individually also induces salt tolerance. Therefore, the present study was conducted to test the hypothesis that supplementation of ethylene to plants receiving N and S under salt stress enhances salt stress tolerance and protects photosynthesis and growth by increasing N and S assimilation and proline metabolism and boosting the antioxidant system.

## 2. Materials and Methods

### 2.1. Plant Materials, Growth Environments, and Treatments

The experiment was set up on mustard (*Brassica juncea* L. Czern & Coss. var. PusaTarak) in the Botany Department of Aligarh Muslim University in Aligarh, India. The seeds were sterilized with HgCl_2,_ washed in distilled water, then grown in 23-cm diameter pots that were filled with a 5 kg soil mix of compost and peat in a 1:4 ratio (*w*/*w*) mixed with sand in a 3:1 ratio (*w*/*w*). Environmental conditions for growing plants in pots were as follows: natural day/night conditions, 640 μmol m^−2^ s^−1^ photosynthetically active radiation (PAR). The day/night temperatures were 24/18 °C (±3 °C) and the relative humidity was 68 ± 5%. Four plants were kept in each pot. In the experiment, ammonium sulphate was the source of N and S. The soil used for the experiment was sandy loam with a pH of 7.4. In the native available soil, N and S were at a concentration of 100 mg kg^−1^ soil each, whereas phosphorus (P) and potassium (K) were at 20 and 90 mg kg^−1^ soil, respectively. The extraction of soil nitrate-N and sulfate was performed with phenol disulphonic acid and calcium chloride, and spectrophotometric and turbidity methods were used for their determination, respectively. Soil P was extracted with sodium bicarbonate and spectrophotometrically determined, while K was extracted with ammonium acetate and estimated using a flame photometer [36,37]. High soil P status was maintained by the addition of 30 mg P kg^−1^ soil as single superphosphate so that this nutrient would not influence the effects of ethephon because upon hydrolysis, ethephon releases ethylene and P. Two plants were maintained per pot, and plants were given deionized water as needed.

N and S were supplemented in split dosages as 50 mg N kg^−1^ soil (N50) plus 50 mg S kg^−1^ soil (S50) at sowing time (0 days), and in similar dosages 20 days after sowing (DAS) [(N50 + S50)_0d_ + _20d_]. Salt stress applications were performed on alternate days at a concentration of 100 mM NaCl for 15 DAS. An amount of 100 mL each of NaCl and distilled water was given alternately for 15 days. To evaluate the role of ethylene in salt stress alleviation through regulation of the assimilation of N and S, proline metabolism, and the antioxidant system, 25 mL of 200 μL L^‒1^ ethephon (Eth; 2-chloroethyl phosphonic acid) as ethylene source was sprayed on the foliage of the control and split N and S receiving plants at 20DAS. An equal amount of the surface active agent Teepol (0.5%; *v*/*v*) was mixed in the treatment of the control and ethephon. The treatments were set in a totally randomized square plan. The number of replicates maintained for every treatment was 4 (*n* = 4). At 40 DAS, key parameters were analyzed.

### 2.2. Oxidative Stress

#### H_2_O_2_ Concentration and Lipid Peroxidation

Oxidative stress level as concentration of H_2_O_2_ and TBARS was measured using the technique of Okuda et al. [38] and Dhindsa et al. [39]. Details are provided in the Supplementary File 1.

### 2.3. Histochemical Staining

The generation of O_2_^•–^ level was analyzed by histochemical staining with nitro-blue tetrazolium chloride (NBT), and the technique of Wang et al. [40] was used to stain the leaves.

### 2.4. Nitrogen Assimilation

#### Activity of Nitrate Reductase and Nitrogen Concentration

The technique of Kuo et al. [41] was used to measure leaf nitrate reductase activity, and N concentration in leaves was computed by the Kjeldahl digestion process as defined by Lindner [42]. The complete process is described in detail in the Supplementary File 1.

### 2.5. Sulfur Assimilation

#### 2.5.1. ATP-sulfphurylase Activity and Sulfur Concentration

Activity of ATP-sulphurylase was examined by the process of Lappartient and Touraine [43]. The turbidimetric procedure of Chesnin and Yien [44] was used for to determine S concentration. The whole procedure is described in the Supplementary File 1.

#### 2.5.2. Cysteine Concentration

Cys concentration in leaves was resolved by the method of Gaitonde [45].

#### 2.5.3. Glutathione Concentration and Redox State

Reduced glutathione (GSH) was evaluated using the method of Anderson [46]. The GSH to GSSG ratio was computed for redox state.

### 2.6. Activities ofAntioxidant Enzymes

Assays for the antioxidant enzymes superoxide dismutase (SOD), catalase (CAT), ascorbate peroxidase (APX), and glutathione reductase (GR) were amplified by the process of Beyer and Fridovich [47] and Giannopolitis and Ries [48], Aebi [49], Nakano and Asada [50] and Foyer and Halliwell [51], respectively. The details are given in the Supplementary File 1.

### 2.7. Proline Metabolism

#### 2.7.1. Estimation of Proline Concentration

Proline concentration in leaf was estimated using the ninhydrin process of Bates et al. [52].

#### 2.7.2. Determination of Glutamyl Kinase and Proline Oxidase Activities

The activities of glutamyl kinase and proline oxidase were examined following the methods of Hayzer and Leisinger [53] and Huang and Cavalieri [54], respectively.

### 2.8. Ethylene Metabolism

#### 2.8.1. ACS Activity

Activity of 1-aminocyclopropane-1-carboxylic acid synthase was measured by the process of Avni et al. [55] and of Woeste et al. [56].

#### 2.8.2. Ethylene

Ethylene evolution was assessed using a method described previously by Fatma et al. [27].

### 2.9. Photosynthetic and Growth Characteristics

Photosynthetic characteristics were examined in fully extended plant leaves by using the Infrared Gas Analyzer (CID-340, Photosynthesis system, Bio-Science, USA) at light saturating intensity at mid-day(PAR: 720 μmol m^−2^ s^−1^) and at 390 ± 15 μmol mol^−1^ atmospheric CO_2_.

Detailed information on maximal PS II photochemical efficiency, chlorophyll concentration, leaf area, and plant dry weight have been given in Fatma et al. [27].

### 2.10. Electron Microscopy

#### Scanning Electron Microscopy

Scanning electron microscopy (SEM) of leaves was performed using the process of Daud et al. [57].

### 2.11. Statistical Analysis

Data were statistically processed using analysis of variance (ANOVA) in SPSS (ver. 17.0 Inc., Armonk, NY, USA) for Windows and presented as means ± standard error (SE). The treatments had 4 sets (*n* = 4). The least significant difference (LSD) was calculated for the significant data at *p* < 0.05. Bars showing the same letter were not significantly different by LSD test at *p* < 0.05.

## 3. Results

### 3.1. Impact of Combined N and S Treatment with Ethephon Supplementation on Photosynthetic and Growth Characteristics under Salt Stress

The growth characteristics were adversely affected by salt stress treatment. Ethephon enhanced leaf area by 34.1% and plant dry mass by 23.5% under no stress, compared to the respective control. Split dosage of N and S also increased leaf area and plant dry mass compared to the control, both under stress and no stress. Under salt stress, split doses of N and S increased leaf area by 73.5% and plant dry mass by 3.3 times compared to salt stress. When Eth was added to NaCl combined with N and S treatments, we observed a greater increase, of 98%, in leaf area, while plant dry mass increased by 3.6 times compared to the plants under salt stress. Eth further enhanced leaf area by 12.5% and plant dry mass by 10.5% when compared to the treatment in which only split doses of N and S were supplied to NaCl-treated plants, suggesting that Eth application to split doses of N and S was better than treatment without Eth (Table 1).

The impact of split dosage of N and S with ethephon on salt-stressed mustard plants were examined by analyzing photosynthetic efficiency. Exogenous application of ethephon exhibited increased PSII efficiency (18.5%), which was lower than split application of N and S. However, under salt stress, both split doses of N and S and Eth alone were equally effective in increasing all photosynthetic parameters and alleviated the negativity of salt stress. However, ethephon with split dosage of N and S (N50 + S50)_0d+20d_ under salt stress was far better than split doses of N and S and significantly improved PSII efficiency by 14.12% compared to when N and S were supplemented without Eth under salt stress (Table 1).

Salt stress decreased Chl concentration, net photosynthesis, stomatal conductance and intercellular CO_2_ concentration. Under no-stress conditions, split doses of N and S were more effective in increasing photosynthetic characteristics than Eth treatment. However, under salt stress, both (either split N and S or Eth) were equally effective. Under salt stress, N and S split application ameliorated the salt toxicity effect and increased Chl concentration by 123.1%, net photosynthesis by 221.5%, stomatal conductance by 1.9 times, and intercellular CO_2_ concentration by 2.2 times compared to salt-treated plants, while a greater increase of 150.6% in Chl concentration, 267.3% in net photosynthesis, 2.1 times in stomatal conductance, and 2.4 times in intercellular CO_2_ concentration was observed with Eth supplementation to N plus S treatment under salt stress. To emphasize the importance of Eth supplementation to N and S treatment, we compared the response of the two treatments. It was notable that when Eth was applied to split N and S treatment, there were significantly larger increases in net photosynthesis by 14.2%, stomatal conductance by 11.1%, intercellular CO_2_ concentration by 10.5%, and chlorophyll content by 12.3%, compared to plants receiving only N and S under salt stress (Table 1).

### 3.2. Oxidative Stress

**Figure 1:**
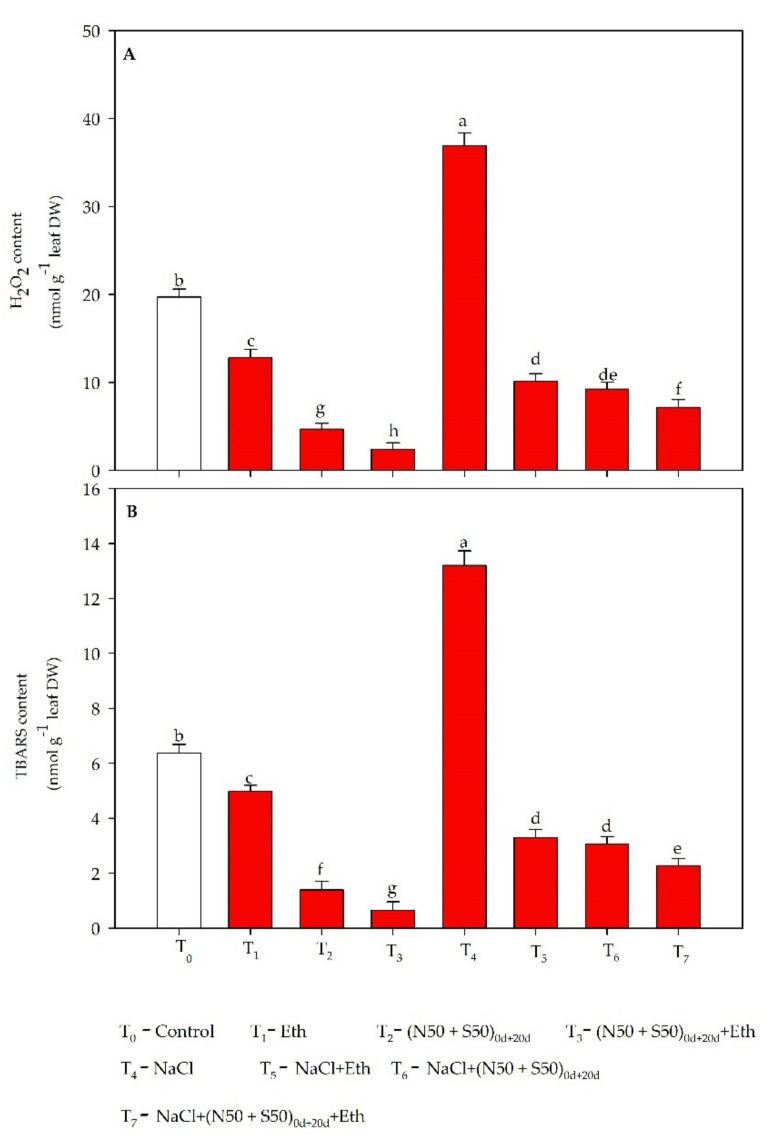
Concentration of (**A**) H_2_O_2_ and (**B**) TBARS in mustard at 40 DAS. Plants were raised with split dosage of N and S (50 + 50 mg kg^−1^ soil) at sowing time (0 DAS) and at 20 DAS, and treated with 200 µL L^−1^ ethephon (Eth) in the absence or presence of 100 mM NaCl. Values are presented as means ± SE (*n* = 4). Data followed by the same letter are not significantly different by LSD test at *p* < 0.05. DAS: days after sowing; DW; dry weight; N: nitrogen; S: sulfur; H_2_O_2_: hydrogen peroxide; TBARS: thiobarbituric acid reactive substances.

Exposure of plants to NaCl showed larger H_2_O_2_ and TBARS concentrations compared to the respective control values. Supplementation of N and S in split doses (N50 + S50) at 0 and then 20 DAS reduced oxidative stress by lowering the H_2_O_2_ concentration by 53.2% and TBARS by 76.7% compared to salt-treated plants. However, we observed that maximum reduction in these parameters occurred when (N50 + S50) at 0 and 20 d were applied in combination with Eth. This significantly decreased H_2_O_2_ and TBARS concentrations, by 22.7 and 22.4%, respectively, compared to the split application of N and S to NaCl-treated plants alone (Figure 1).

### 3.3. ROS Accumulation

**Figure 2:**
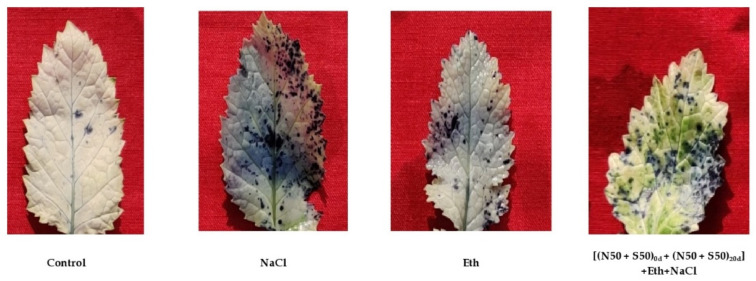
In situ determination of generated levels of superoxide ion (O_2_^•−^) through nitro blue tetrazolium staining in mustard after dehydration of leaves at 40 DAS. Plants were raised with 0.1 mM NaCl alone or in combination with 200 µL L^−1^ ethephon (Eth) with split dosage of N and S [(N50 + S50)_0d_ + _20d_].

Salt stress brought about ROS overproduction in plants. The enhanced concentration of ROS led to oxidative damage in the mustard plants, with increased lipid peroxidation and damage to cell membranes. The generatedO_2_^•−^ level in leaves was assessed using NBT as a histochemical process. The level of generated O_2_^•−^ was viewed through blue staining 6 h after treatment of leaves with NBT. The stained spots were more conspicuous in salt-treated leaf discs, compared to control leaves. Ethephon in the salt-treated plants decreased the O_2_^•−^ spots, compared to plants receiving the salt-alone treatment. Plants with ethephon added to split dosage of N and S showed only limited staining under salt treatment, compared to the NaCl-treated plants (Figure 2).

### 3.4. Antioxidant Enzymes and Salt Tolerance

Salt-treated plants increased antioxidant enzyme activity compared to the control values. Ethephon and split applications of N and S under salt stress were equally effective in increasing CAT, APX, GR, and SOD activity compared to the control. However, it was the combined Eth treatment with split N and S application to salt-treated plants that was more effective in increasing antioxidant enzyme activity to overcome the oxidative stress. Ethephon supplementation to plants receiving salt and (N50 + S50) at 0 and 20d increased activity of SOD by 12.5%, CAT by 14.0%, APX by 14.8%, and GR by 15.5% in comparison to the plants that received only split doses of N and S under salt stress, emphasizing the importance of Eth with split doses in salt tolerance (Table 2).

### 3.5. Nitrogen and SulfurAssimilation

The importance of N and S or ethephon in N assimilation was analyzed by studying NR activity and N concentration. S assimilation was analyzed by studying ATP-S activity, concentration of S, Cys, and GSH, and redox state. The treatment of 100 mM NaCl reduced N and S concentration and redox state but increased the ATP-S activity and concentration of Cys and GSH compared to the respective control values. Activity of NR was reduced under salt stress. Ethephon application markedly improved the concentration of N by 16.6% and NR activity by 41.8% compared to the respective control values. Application of the split form of N and S (N50 + S50) at 0d and 20d enhanced N concentration and the activity of NR strikingly under stress or without stress. Under non-stress conditions, it was better than Eth treatment, whereas under salt stress it was equally as effective as ethephon. The split doses of N and S also alleviated the stress; however, when compared to the addition of Eth treatments to split N and S, we observed a much greater increase in NR activity and the concentration of N, which was above that from the split doses, suggesting that Eth enhanced N assimilation under salt stress. Eth with split N and S increased the concentration of N by 15.0% and NR activity by 10.3% compared to the split N and S treatment only.

Application of ethephon with split N and S under salt stress increased ATP-S activity and S, Cys, and GSH concentrations by 14.1%, 13.3%, 13.6%, and 15.6%, respectively, and the redox ratio by 10.8% in comparison to plants that received only split doses of N and S under salt stress. Thus, the combination of ethephon and split dosage of N and S (N50 + S50)_0d + 20d_ in salt-treated plants increased N and S assimilation more prominently and substantially than split doses of N and S alone under salt stress (Table 3).

### 3.6. Proline Metabolism under Salt Stress

Salt-stressed plants showed a significant response in terms of proline metabolism. Proline accumulation increased in salt-stressed plantswith the supplementation of combined dosage of N and S compared to the control plants. Salt treatment increased proline concentration by 22.9% compared to the respective control plants. Individual application of ethephon considerably increased proline accumulation by 47.8%, and split N and S (N_50_ + S_50_) and 20d increased proline concentration by 60.9% compared to the respective control plants. Plants receiving ethephon under salt stress increased proline accumulation considerably and significantly equal to plants that received split doses of N and S. However, ethephon together with the split doses of N and S further increased the concentration of proline both under stress and no stress. Under salt stress, it was found to be better than N and S treatment and increased proline concentration by 17.7% compared to split N and S treatment under salt stress (Table 4).

Application of ethephon individually enhanced glutamyl kinase (GK) activity by 35.4% compared to control plants. Plants receiving split doses of N and S in the absence or presence of salt improved GK activity compared to the respective control. However, it was ethephon application under salt stress together with the split doses of N and S that more conspicuously increased GK activity, by 63.3% compared to a 46.8% increase obtained with split doses of N and S under salt stress, and when compared to salt treated plants. Eth with split N and S yielded an 11.6% increase in GK activity over the treatment with only split N and S doses under salt stress. The split doses of N and S with ethephon yielded a maximum reduction in proline oxidase (PROX) activity compared to the respective control (Table 4).

### 3.7. Ethylene Biosynthesis under Salt Stress

**Figure 3:**
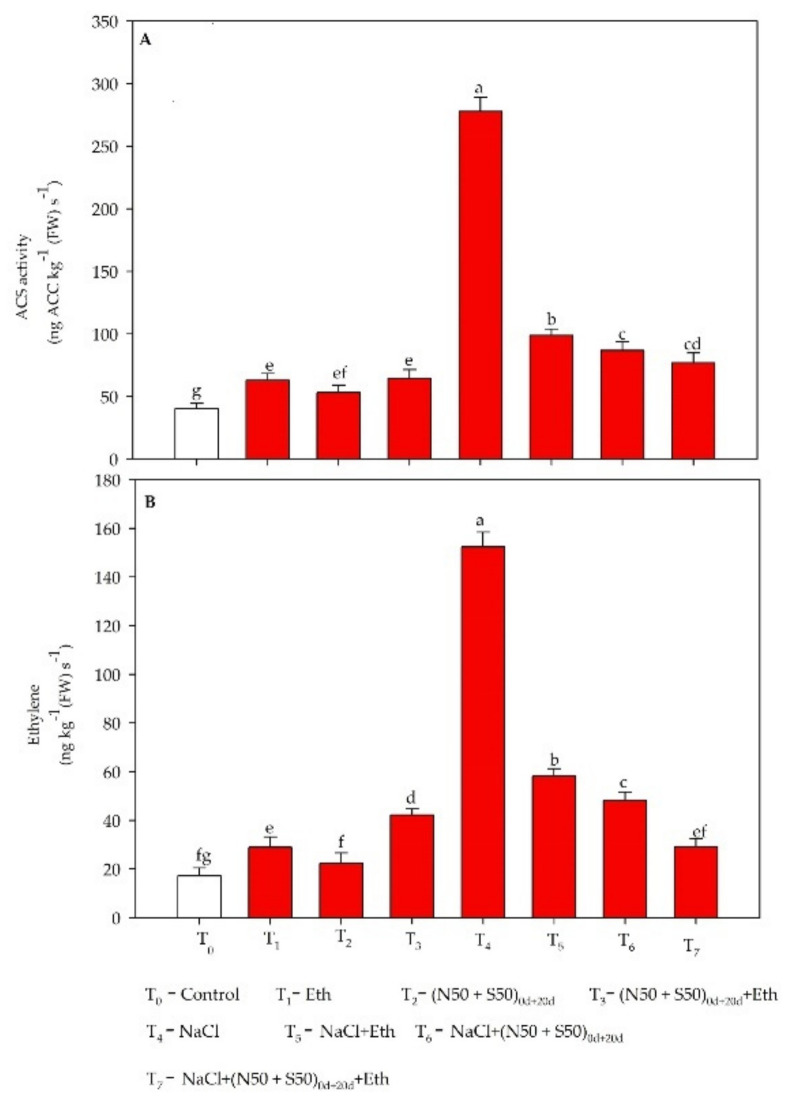
(**A**) Activity of 1-aminocyclopropane carboxylic acid synthase (ACS) and (**B**) ethylene production in mustard plants at 40 DAS. Plants were raised with split doses of N and S (50 + 50 mg kg^−1^ soil) at sowing time (0 DAS) and at 20 DAS, and were treated with 200 µL L^−1^ ethephon (Eth) in the absence or presence of 100 mM NaCl. Values are presented as means ± SE (*n* = 4). Data followed by the same letter are not significantly different by LSD test at *p* < 0.05. DAS: days after sowing; FW: fresh weight.

Salt-grown plants exhibited maximum ethylene production and ACS activity by 8.9- and 6.9-fold compared to the respective control values.ACS activity and ethylene production increased with supplementation of Eth to split doses of N and S under no stress. Supplementation of split doses of N and S or Eth equally decreased the stress ethylene level and reduced it below the level in salt-stressed plants. However, plants grown with Eth under salt stress with split doses of N and S decreased ACS activity by 72.3% and ethylene production by 80.9% compared to the salt-treated plants (Figure 3). In comparison to the treatment with salt+ N and S, Eth with split doses of N and S decreased ACS activity by 11.2% and ethylene evolution by 39.6% under salt stress.

### 3.8. Stomatal Behavior under Salt Stress

**Figure 4:**
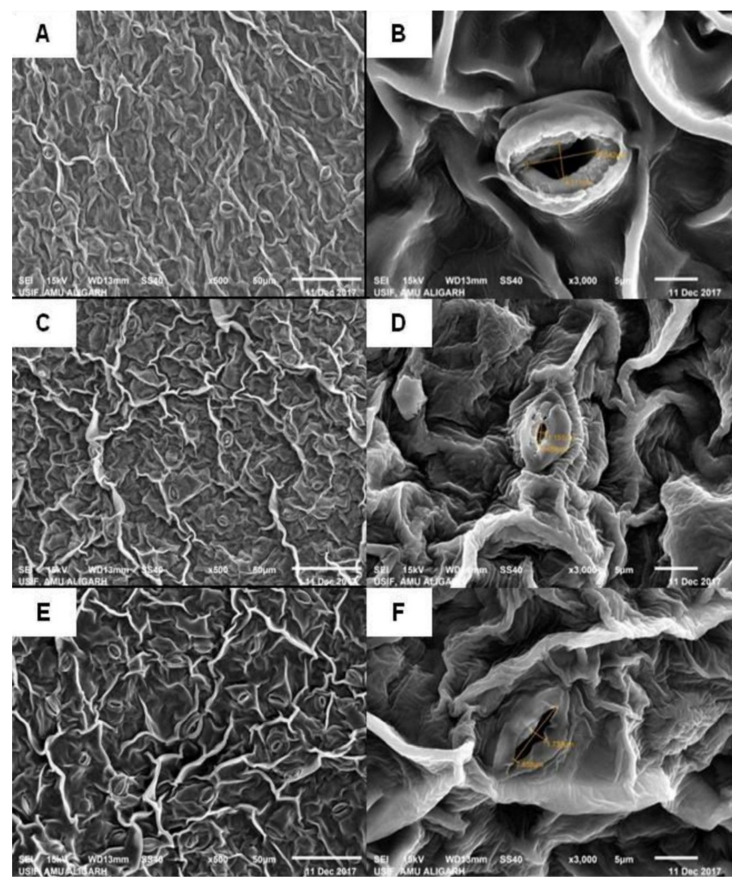
Stomatal behavior of mustard examined under (**A**,**B**) control, (**C**,**D**) 100 mM NaCl, and (**E**,**F**) ETH treatment with split doses of N and S with 100 mM NaCl. The stomatal response of opening and closing was viewed with SEM at 500 × (**A**,**C**,**E**) and 3000 × (**B**,**D**,**F**) magnification in mustard plants developed under 100 mM NaCl at 40 DAS. Bars (**A**,**C**,**E**) = 50 µm; bars (**B**,**D**,**F**) = 5 µm.

Stomatal response was studied in the presence of ethephon supplementation with or without split doses of N and S in the presence or absence of salt. The width and length of stomata were 4.1 and 8.3 µm in the control plants. SEM analysis showed slight closing of the stomatal pores under salt stress, and the frequency of stomata decreased by 10.5% compared to respective control plants. The application of exogenous ethephon with split doses of N and S led to improved width and length of stomata by 1.7 and 7.5 μm, respectively, and the frequency of stomata by 42.1% compared to control plants values. The application of ethephon with split doses of N and S under stress or no stress led to higher number stomatal frequency compared to control plants (Figure 4).

## 4. Discussion

### 4.1. Ethephon Plus N and S Mediated Oxidative Stress and Activity of Antioxidant System under Salt Stress

Salt stress leads to an increase in oxidative stress and decrease in the uptake and assimilation of nutrients due to excess accumulation of Na^+^ ions. The studies showed that the tolerance of plants was induced using exogenous plant hormone, nutrients, and antioxidant systems, via balancing homeostasis of ions [6,27,58]. The present study gained novelty by using exogenous ethephon with split doses of N and S in salt-stressed plants that increased the antioxidant defense system and substantially decreased H_2_O_2_ and TBARS concentrations.

The production of ROS and excessive accumulation under salt stress caused the maximum damage in the plant cells. It was interesting to note that decreased oxidative stress in plants treated with Eth combined with N and S corresponded with huge up-regulation of the cell antioxidative system. Activity of APX, SOD, CAT, and GR was improved under conditions of salt stress, possibly to neutralize the salt stress-induced oxidative damage. Plants enhance their antioxidant system to counter the harmful impact of abiotic stress [11,26,59,60]. The enzymatic and non-enzymatic antioxidants work in cells to neutralize oxidative stress and to keep their concentration levels below toxic levels [61,62]. Although, declines in oxidative stress induced by Eth or individual supplementation of N and S have been previously reported, their interactive role has received scant attention. The present study reported that decreased rates of ROS accumulation in plants treated with Eth combined with N and S may have directly provided protection to cellular organelles and thus to their smooth function. This report is supported by earlier studies in which applications of Eth reduced oxidative stress and increased antioxidant enzyme activity in *B. juncea* [59,63,64]. The exogenous application of Eth combined with split doses of N and S caused minimum staining due to enhanced assimilation of N and S and synthesis of GSH, proline, and antioxidant enzymes.

The present study showed that more promising results in enhancing the antioxidant system and lowering oxidative stress were obtained when Eth and N and S were applied together. It is likely that Eth combined with split doses of N and S lessened the oxidative stress in treated plants more efficiently than did separate applications of Eth or N and S. It is conceivable that exogenous Eth with split doses of N and S mitigated ROS-arbitrated oxidative stress by activating antioxidant enzymes.

### 4.2. Nitrogen- and Sulfur-Induced Increase in N and S -Assimilation, Proline and GSH Concentration Enhanced Further with Ethylene

Nitrogen metabolism is vital to the development of plants and plays a key role in environmental fluctuations [6,65]. Salt stress impairs the uptake and distribution of nutrients due to its direct and indirect effect on N metabolism [66]. In the present study, we observed decreased NR activity and N concentrations under salt stress. Photosynthetic efficiency was directly related to decreased NR activity, and any decrease in nitrate assimilation was accountable for the decreased admittance of CO_2_, causing inactivation of the NR enzyme [67]. However, exogenous application of Eth plus N and S showed a positive impact on N assimilation and NR activity in NaCl-treated plants. Ethylene application enhances N metabolism and increases NR activity and N concentrations in *B. juncea* [68]. Besides increased N assimilation, Eth in plants supplemented with N and S in split doses increases proline concentration under salt stress. Increases in proline with N assimilation have been reported under salt stress [69,70]. Proline functions as an antioxidant and scavenges ROS to protect plants from oxidative stress [71,72]. Maximum proline accumulation via increases in its biosynthesis enzyme and decreases in its degradation enzyme suggest its role in defending plants against salt stress. Proline accumulation increased maximally with Eth and N and S application under stress and helped in stress alleviation. Reports suggest proline accumulation helps overcome stressful environments, where it improves PSII electron transport [73] and regulates the redox potential of cells together with its role in increasing N remobilization and N use-efficiency [74,75,76]. Thus, in our study, the raised proline concentration due to salt stress may be a direct result of increased proline biosynthesis, with a decline in its oxidation to maintain water balance. Eth application induced GK activity and inhibited POX activity to enhance proline accumulation. The activity of P5CS and GK has a vital role in regulating the proline level and abiotic stress in plants [77]. The increment in proline metabolism was related to ethylene levels in plants under salt stress and in turn to the N level [21]. The present study suggested that Eth application with split doses of N and S maximally improved proline metabolism and added to salt tolerance with increased photosynthetic efficiency through reduced oxidative stress and by maintaining water relations as an osmolyte. Proline maintains osmotic adjustments of the salt-treated plants to provide optimal conditions for cellular reactions. Thus, based on reported studies, we can suggest that Eth application to plants receiving N and S helped in increasing N assimilation, which subsequently was invested in enhanced proline accumulation to enhance salt tolerance.

Similarly to N assimilation, the assimilation of S prompts GSH production and alleviates the injurious influence of salt stress. Exogenous application of Eth to plants in salt stress significantly enhanced S and Cys concentrations with GSH and activity of ATP-S, which improved the redox status of the cellular environment in the present study. A related study of involving *B. juncea* has revealed that Eth increases ATP-sulfurylase activity and S accumulation [78]. Reports on the effect of Eth with split N and S doses on S-containing amino acids and reduced thiols (Cys, GSH) under salt stress are scarce in the literature. Although no direct reports are available, individually Eth influences N and S assimilation and GSH content, which is also affected by N and S supplementation [79]. The application of Eth increased the photosynthetic performance of salt-treated plants through improved thiol compounds and then led to a greater redox ratio in the presence or absence of stress with split doses of N and S. Cysteine is accountable for production of several important S-containing compounds, such as methionine and GSH [80,81]. Besides this, the accumulation of GSH depends on Cys availability through S assimilation under salt stress conditions [5,82]. Therefore, Cys biosynthesis in plants is a critically important metabolic process that allows and serves as the branch point between S and N assimilation, as the carbon skeleton and amino group of Cys are derived from serine, a product of N assimilation [82]. Furthermore, ATP-S, the first enzyme in the S assimilation pathway, regulates the synthesis of S compounds. Thus, enhanced ATP-S activity can develop plant tolerance through increased thiol compoundconcentrations under abiotic stress, which helps in the removal of excess ROS [63]. This study suggests that application of Eth is associated with S assimilation via Cys; it is expected that S affects ET sensitivity, and ET is involved in regulating GSH production and salt stress alleviation. In the previous study on *B. juncea* plants, it was suggested that the application of Eth increased the concentration of reduced thiols such as Cys and GSH and improved photosynthesis and growth under Cr stress [83]. GSH production is well-known for the removal of excess ROS and protects plants from oxidative damage [84,85,86]. The homeostasis of GSH and GSSG maintains signaling of stress-responsive proteins and regulates oxidative stress. The increase in GSH and redox state with S and Eth has been suggested [27]. Thus, here we can assume that Eth applications combined with N and S could enhance their assimilation, leading to increased N concentrations, NR activity, proline concentrations, and GSH concentrations to reduce oxidative stress.

### 4.3. Influence of Eth with Supplementation of N and S on Ethylene Biosynthesis

Adverse environmental conditions cause rapid ethylene emission, which is called stress ethylene. The biosynthesis of ethylene occurs through simple metabolic pathways [87,88,89], and ACC oxidase and ACC synthase are the two important enzymes involved in ethylene production. Study of the ethylene biosynthetic pathway has shown that enzymes and the genes encoding those enzymes are responsible for the rapid production of ET under stress conditions [87,88,90,91]. Salt stress enhanced ethylene biosynthesis but this is stress ethylene and it has to be brought down to an optimum level that favors plant photosynthesis and growth. Increased ET production under abiotic stress induces oxidative stress and affects photosynthetic processes [92]. The increase in ethylene sensitivity with S under Cd stress has been reported [59]. Similarly, N supplementation decreased stress ethylene evolution under stress and increased ethylene-mediated responses [21]. In the present study, we observed that in the presence of both N and S, when Eth is applied we obtain optimum ethylene.

### 4.4. Ethylene Supplementation with N and S Improved Photosynthetic and Growth Performance under Salt Stress

Modification of photosynthetic efficiency under environmental stress is crucial for plant persistence [93], and plants accept this scheme by moderating stomatal conductance and intercellular CO_2_ concentration in addition to protecting chlorophyll content [5,27]. To explain the salt stress effects and interaction with ET and N and S on photosynthesis, we investigated photosynthesis-related parameters.

Exogenous application of Eth enhances photosynthetic capacity in *B. juncea* [25,27,59,63,64] under abiotic stress by increasing stomatal conductance and the activity of Rubisco [64,78]. Application of Eth increased photosynthesis due to an increased diffusion rate of CO_2_ through intercellular spaces and increases in photosynthetic pigments and stomatal aperture [27]. The application of Eth with split doses of N and S accomplished more in augmenting photosynthetic capacity under salt stress. The split doses of N and S at the two developmental stages of plants, one at the time of sowing and the other at 20 DAS, facilitated better N and S assimilation and the formation of reduced S compounds through an enhanced antioxidant system. These processes were favored by ethylene due to Eth application. This led to protection of the photosynthetic machinery from salt-induced oxidative stress. The positive mechanisms of ET along with N and S on photosynthesis accounted for the protection of chlorophyll, decreased ROS, and increased antioxidant enzyme activity under salt stress. It has been shown that N availability affects ET evolution and the efficiency of stomatal conductance and photosynthesis [21].

The results also showed that salt stress decreases chlorophyll content, which agrees with previous reports for *Vigna radiata* [65] and *Medicago sativa* seedlings [94]. Application of Eth plus N and S increased chlorophyll content and led to active photosynthetic activity, causing growth. Photosynthesis is directly related to crop production under several metabolic processes [93]; an ineffective photosynthetic capacity will lead to yield loss under stress [94].

The study of Chl fluorescence may highlight the impact on plants in stressed conditions, focusing on the potentiality of plants in environmental fluctuation [95,96]. The ratio *F*_v_/*F*_m_ denotes the photosynthetic efficiency of whole PSII and the maximum quantum yield of PSII [97]. The present study has shown that salt treatment reduced *F*_v_/*F*_m,_ and the decrease in photosynthesis was primarily due to photo-inhibition under salt stress. Exogenous Eth plus N and S limited the decrease in photosynthetic efficiency and averted salt stress-induced photo inhibition.

The application of Eth combined with doses of N and S clearly enhanced thiol production, which provided the most protection to the photosynthetic system and consequently to leaf area and plant dry mass in salt-treated plants. Ethylene mitigated salttoxicity in *Medicago sativa* by reducing oxidative stress [94]. Moreover, the higher leaf area is related to ethephon-improved ET synthesis [28,98]. Earlier, it was reported that Eth application to *Arabidopsis* and *Nicotiana tabacum* increased leaf area, plant dry weight, and pod number at low concentrations, but inhibited these at high concentrations [99].Studies have showed that ET cooperates with nutrient uptake and regulates plant responses under stress for improvements in plant dry weight and leaf area [11,28,63].

### 4.5. Ethylene with N and S Modulated Stomatal Behavior under Salt Stress

Stomata are minute apertures in plant leaves that stimulate gas and water exchange among the plant and its related environs. Consequently, the closing and opening of the stomatal aperture is a major characteristic of maintaining the transpiration rate and photosynthesis [100]. Salt treatment induced partial stomatal closure; because of the excessive accumulation of ions, the guard cells became flaccid. However, the stomata were found to be open in plants receiving Eth with N and S. The application of Eth plus N and S highly influenced the osmotic relations that resulted in stomatal opening. Furthermore, studies have shown ET to be involved in both stomatal opening and closure [101], depending on the situation. Additionally, the plant hormone abscisic acid (ABA) plays a central function in directing stomatal closure through the synthesis of second messengers, which involves ROS accumulation. Some studies report ET-induced stomatal closure through NADPH oxidase-mediated ROS accumulation in the guard cells [102,103]. Further, another pathway involving EIN2 showed negative regulation of stomatal closure. Ethylene treatment interacted with ABA to influence stomatal closure [104]. It has been shown that flavonols accumulate in guard cells because of ET activity [105]. These flavonols inhibit ROS production and stomatal closure. Ethylene and its precursor ACC stimulate H_2_O_2_ accumulation in guard cells and cause the closure of the stomatal aperture in *Vicia faba* [106].

## 5. Conclusions

Supplemental N and S applied in split doses have a positive effect in mustard plants and alleviate the negative effects of salt stress. Salt stress remarkably decreased the photosynthetic efficiency of plants by increasing ROS generation. Both split application of N and S and ethephon were effective in combating salt stress. However, ethephon with N and S mitigated the salt stress-induced damaging effects and improved plant growth by up-regulating antioxidant enzyme activity and the accumulation of osmolytes more conspicuously than split N and S treatment alone. This increased tolerance with comparatively better growth under salt stress as a result of ethephon application coupled with N and S could be attributed to increased N and S assimilation when they are already available to the plants. Ethylene in plants under salt stress played a noteworthy role in N and S-mediated regulation of photosynthetic and growth characteristics by increasing their availability for proline and GSH formation. In light of the results obtained, we can infer that the inhibitory effects of salt on photosynthesis and growth were reversed significantly when ethephon plus N and S were applied together, via reduced salt-induced ROS production and increased N and S assimilation. This study could be exploited to enhance nutrient utilization through the supplementation of ethephon as source of ethylene for enhancement of growth under both normal and stress conditions.

## Figures and Tables

**Table 1:** Net photosynthesis, chlorophyll content, stomatal conductance, intercellular CO_2_ concentration, leaf area and plant dry mass in mustard at 40 DAS. Plants were raised with split dosage of N and S (50 + 50 mg kg^−1^ soil) at sowing time (0 DAS) and at 20 DAS, and plants were treated with 200 µL L^−1^ ethephon (Eth) in the absence or presence of 100 mM NaCl. Values are presented as means ± SE (*n* = 4). Data followed by the same letter are not significantly different by LSD test at *p* < 0.05. DAS: days after sowing.

Treatments	Net Photosynthesis (μmol CO_2_ m^−2^ s^−1^)	Chlorophyll Content (SPAD Value)	Stomatal Conductance (mmol CO_2_ m^−2^ s^−1^)	Intercellular CO_2_ Concentration (μmol CO_2_ mol^−1^)	Maximum Efficiency of PSII	Leaf Area (cm^2^ plant^−1^)	Plant Dry Mass (g plant^−1^)
Control	14.24 ± 0.36 ^f^	28.22 ± 0.71 ^e^	368.17 ± 9.26 ^e^	272.11 ± 6.85 ^f^	0.65 ± 0.0099 ^e^	129.26 ±3.24 ^f^	2.34 ± 0.06 ^f^
Eth	21.71 ± 0.55 ^e^	37.66 ± 0.95 ^d^	462.12 ± 10.60 ^d^	342.36 ± 8.61 ^e^	0.77 ± 0.0121 ^d^	173.64 ±4.35 ^e^	2.89 ± 0.07 ^e^
(N50 + S50)_0d + 20d_	30.71 ± 0.47 ^b^	48.85 ± 0.75 ^b^	609.54 ± 12.40 ^b^	448.55 ± 6.84 ^b^	0.95 ± 0.0148 ^b^	227.05 ±3.46 ^b^	3.77 ± 0.06 ^b^
(N50 + S50)_0d + 20d_ + Eth	33.46 ± 0.51 ^a^	52.71 ± 0.81 ^a^	643.56 ± 13.82 ^a^	476.94 ± 7.27 ^a^	1.02 ± 0.0156 ^a^	239.51 ±3.65 ^a^	4.02 ± 0.06 ^a^
NaCl	07.93 ± 0.32 ^g^	19.25 ± 0.77 ^f^	289.43 ± 9.12 ^f^	178.12 ± 7.12 ^g^	0.49 ± 0.0196 ^f^	062.53 ±2.5 ^g^	1.02 ± 0.04 ^g^
NaCl + Eth	24.25 ± 0.49 ^d^	41.67 ± 0.69 ^c^	529.26 ± 10.14 ^c^	386.16 ± 7.8 ^d^	0.83 ± 0.0165 ^c^	189.24 ± 3.86 ^d^	3.28 ± 0.06 ^d^
NaCl + [(N50 + S50)_0d + 20d_]	25.50 ± 0.53 ^d^	42.95 ± 0.89 ^c^	537.71 ± 11.10 ^c^	392.21 ± 8.16 ^d^	0.85 ± 0.0177 ^c^	198.82 ±4.12 ^d^	3.32 ± 0.07 ^d^
NaCl + [(N50 + S50)_0d + 20d_] + Eth	29.13 ± 0.61 ^c^	48.25 ± 1.00 ^b^	597.22 ± 12.30 ^b^	433.27 ± 8.81 ^c^	0.97 ± 0.0193 ^b^	223.68 ±4.47 ^c^	3.67 ± 0.07 ^c^

**Table 2:** The antioxidant enzyme activity of superoxide dismutase (SOD), catalase (CAT), ascorbate peroxidase (APX), and glutathione reductase (GR) reduced glutathione (GSH) content and redox state in mustard at 40 DAS. Plants were raised with split dosage of N and S (50 + 50 mg kg^−1^ soil) at sowing time (0 DAS) and at 20 DAS and treated with 200 µL L^−1^ ethephon (Eth) in the absence or presence of 100 mM NaCl. Values are presented as means ± SE (*n* = 4). Data followed by the same letter are not significantly different by LSD test at *p* < 0.05. DAS: days after sowing.

Treatment	SOD Activity	CAT Activity	APX Activity	GR Activity
	(U mg^−1^Protein min^−1^)			
Control	6.58 ± 0.16^f^	118.01 ± 2.96^f^	1.32 ± 0.03^h^	0.192 ± 0.005^f^
Eth	12.20 ± 0.30^d^	153.43 ± 3.86^d^	2.56 ± 0.06^f^	0.277 ± 0.007^d^
(N50 + S50)_0d + 20d_	17.70 ± 0.27^b^	207 02 ± 3.16^b^	4.37 ± 0.06^b^	0.397 ± 0.006^b^
(N50 + S50)_0d + 20d_ + Eth	19.20 ± 0.29^a^	232.07 ± 3.54^a^	4.62 ± 0.07^a^	0.428 ± 0.007^a^
NaCl	8.80 ± 0.35^e^	139.00 ± 5.56^e^	1.85 ± 0.07^g^	0.230 ± 0.009^e^
NaCl + Eth	14.78 ± 0.26^c^	164.13 ± 2.14^cd^	3.34 ± 0.05^de^	0.319 ±0.006^c^
NaCl + (N50 + S50)_0d + 20d_	15.50 ± 0.32^c^	178.03 ± 3.70^c^	3.57 ± 0.07^d^	0.329 ± 0.006^c^
NaCl + (N50 + S50)_0d +20d_+ Eth	17.03 ± 0.36^b^	203.07 ± 4.22^b^	4.10 ± 0.08^c^	0.380 ± 0.008^b^

**Table 3:** Activity of nitrate reductase (NR) and ATP-sulfurylase (ATP-S), content of nitrogen (N) and sulfur (S), cysteine (Cys), reduced glutathione (GSH), and redox state in mustard plants at 40 DAS. Plants were raised with split dosage of N and S (50 + 50 mg kg^−1^ soil) at sowing time (0 DAS) and at 20 DAS, and treated with 200 µL L^−1^ ethephon (Eth) in the absence or presence of 100 mM NaCl. Values are presented as means ± SE (*n* = 4). Data followed by the same letter are not significantly different by LSD test at *p* < 0.05. DAS: days after sowing; DW: dry weight.

Treatments	NR Activity (nmol NO_2_ h^−1^)	ATP-S Activity (µmol g^−1^ Protein s^−1^)	N Content (mg g^−1^ DW)	S Content (mg g^−1^ DW)	Cys Content (nmol g^−1^ Leaf DW)	GSH Content (nmol g^−1^ Leaf DW)	Redox State (GSH/GSSG)
Control	414.35 ± 10.40 ^e^	1.36 ± 0.03 ^f^	35.91 ± 0.90 ^f^	4.83 ± 0.12 ^e^	6.41 ± 0.16 ^f^	65.13 ± 1.64 ^g^	17.91 ± 0.45 ^f^
Eth	587.48 ± 14.84 ^d^	2.65 ± 0.07 ^d^	41.87 ± 1.05 ^e^	6.10 ± 0.15 ^d^	8.46 ± 0.21 ^d^	87.25 ± 2.20 ^e^	25.43 ± 0.63 ^e^
(N50 + S50)_0d + 20d_	739.48 ± 11.36 ^b^	3.62 ± 0.05 ^b^	54.98 ± 0.84 ^b^	8.57 ± 0.13 ^b^	11.67 ± 0.17 ^b^	126.50 ± 1.92 ^b^	36.76 ± 0.56 ^b^
(N50 + S50)_0d + 20d_ + Eth	784.53 ± 11.91 ^a^	3.83 ± 0.05 ^a^	58.94 ± 0.89 ^a^	9.28 ± 0.14 ^a^	12.78 ± 0.19 ^a^	134.34 ± 2.04 ^a^	38.76 ± 0.59 ^a^
NaCl	325.27 ± 13.03 ^f^	1.59 ± 0.06 ^e^	23.85 ± 0.95 ^g^	3.83 ± 0.15 ^f^	7.44 ± 0.29 ^e^	79.27 ± 3.18 ^f^	08.42 ± 0.33 ^g^
NaCl + Eth	632.12 ± 12.46 ^c^	3.03 ± 0.05 ^c^	47.11 ± 0.99 ^d^	7.31 ± 0.14 ^c^	9.79 ± 0.25 ^c^	97.14 ± 1.97 ^d^	28.1 ± 0.31 ^d^
NaCl + (N50 + S50)_0d + 20d_	652.84 ± 13.66 ^c^	3.13 ± 0.06 ^c^	48.24 ± 1.01 ^d^	7.43 ± 0.15 ^c^	9.88 ± 0.20 ^c^	102.25 ± 2.12 ^d^	30.55 ± 0.63 ^d^
NaCl + (N50 + S50)_0d + 20d_ + Eth	713.03 ± 14.81 ^b^	3.57 ± 0.07 ^b^	51.84 ± 1.07 ^c^	8.42 ± 0.17 ^b^	11.23 ± 0.23 ^b^	118.18 ± 2.45 ^c^	34.27 ± 0.71 ^c’^

**Table 4:** The activity of glutamyl kinase (GK), proline oxidase (PROX), and the content of proline in mustard plants at 40 DAS. Plants were raised with split doses of N and S (50 + 50 mg kg^−1^ soil) at sowing time (0 DAS) and at 20 DAS, and were treated with 200 µL L^−1^ ethephon (Eth) in the absence or presence of 100 mM NaCl. Values are presented as means ± SE (*n* = 4). Data followed by the same letter are not significantly different by LSD test at *p* < 0.05. DAS: days after sowing.

Treatments	GK Activity (U mg^−1^ Protein min^−1^)	PROX Activity (U g^−1^ Protein min^−1^)	Proline Content (mg g^−1^ FW)
Control	0.65 ± 0.02 ^h^	90.05 ± 2.04 ^a^	05.63 ± 0.14 ^g^
Eth	0.88 ± 0.03 ^f^	53.07 ± 1.08 ^c^	8.32 ± 0.26 ^e^
(N50 + S50)_0d + 20d_	0.96 ± 0.02 ^e^	29.04 ± 0.41 ^d^	9.06 ± 0.27 ^d^
(N50 + S50)_0d + 20d_ + Eth	1.01 ± 0.03 ^d^	25.00 ± 0.40 ^e^	10.12 ± 0.33 ^b^
NaCl	0.79±0.04 ^g^	61.05±1.44 ^b^	06.92±0.35 ^f^
NaCl + Eth	1.12 ± 0.05 ^bc^	06.10 ± 0.14 ^f^	9.32 ± 0.42 ^c^
NaCl + (N50 + S50)_0d + 20d_	1.16 ± 0.04 ^b^	06.21 ± 0.12 ^f^	9.48 ± 0.40 ^c^
NaCl + (N50 + S50)_0d + 20d_ + Eth	1.29 ± 0.05 ^a^	05.23 ± 0.01 ^g^	11.16 ± 0.48 ^a^

## Data Availability

The data presented in this study are available in the graphs and tables provided in the manuscript.

## Supplementary Materials

The following is available online at https://www.mdpi.com/article/10.3390/plants10071303/s1, Supplementary File 1: Details on methodology.
