# Supplementary material for: Ethylene Supplementation Combined with Split Application of Nitrogen and Sulfur Protects Salt-Inhibited Photosynthesis through Optimization of Proline Metabolism and Antioxidant System in Mustard (Brassica juncea L.)
Source: Plants (Basel). 2021 Jun 27;10(7):1303. doi: 10.3390/plants10071303 (PMC8309136; doi:10.3390/plants10071303)
Supplement: Supplementary file 1 [file plants-10-01303-s001.zip › plants-1264882-supplementary.pdf]

Supplementary file for material and methods

Ethylene Supplementation Combined with Split Application of Nitrogen and Sulfur Protects Salt-Inhibited Photosynthesis through Optimization of Proline Metabolism and Antioxidant System in Mustard (*Brassica juncea* L.)

Badar Jahan[1†], Noushina Iqbal[2†], Mehar Fatma[1], Zebus Sehar[1], Asim Masood[1], Adriano Sofo[3,*], Ilaria D'ippolito[3], Nafees A. Khan[1,*]

1 Plant Physiology and Biochemistry Laboratory, Department of Botany, Aligarh Muslim University, Aligarh-202002, India; naziabadar.2014@gmail.com (B.J.); naushina.iqbal@gmail.com (NI); meharfatma30@gmail.com (M.F); seharzebus5779@gmail.com (Z.S); asim.bot@gmail.com (A.M.); naf9.amu@gmail.com (N.A.K.)

2 Department of Botany, Jamia Hamdard, New Delhi-10062, India

3 Department of European and Mediterranean Cultures: Architecture, Environment, Cultural Heritage (DiCEM), University of Basilicata, 75100 Matera, Italy; adriano.sofo@unibas.it (A.S); dippolito.ilaria@libero.it (I.D.)

* Corresponding authors: naf9.amu@gmail.com (N.A.K); adriano.sofo@unibas.it (A.S.)

† Authors share first authorship

2. Material and Methods

2.2. Oxidative stress

2.2.1. Determination of H_2O_2 content and lipid peroxidation

H_2O_2 content was determined following the method of Okuda et al. (1991). Fresh leaf tissues (500 mg) were ground in ice-cold 200mM $HClO_4$. Homogenized samples were centrifuged at 1200 x *g* for 10 min. After that, perchloric acid of the supernatant was neutralized with 4M KOH. Homogenized material was further centrifuged at 500 x *g* for 3 min for the elimination of insoluble $KClO_4$. The final reaction mixture volume (1.5 ml) consisted of 1 ml of the eluate, 80 µl of 3-methyl-2-benzothiazoline hydrazone, 400 µl of 12.5mM 3-(dimethylamino) benzoic acid in 0.375M phosphate buffer (pH 6.5), and 20 µl of peroxidase (0.25 unit). The reaction was started with the addition of peroxidase at 25°C. The increase in absorbance was estimated at 590nm on a spectrophotometer (5430R Eppendorf). The H_2O_2 content was calculated using the extinction coefficient 0.28 $\mu mol^{-1}cm^{-1}$.

Lipid peroxidation in leaves was measured following the method of Dhindsa et al. (1981) by estimating the content of thiobarbituric acid (TBA) reactive substance (TBARS). Fresh leaf samples (500 mg) were homogenized in 0.25% 2-TBA in 10% trichloroacetic acid (TCA) using mortar and pestle. At 95°C, homogenized samples were heated for 30min and then rapidly cooled in an ice bath and centrifuged at 10000 x *g* for 10min. To 1 ml aliquot of the supernatant, 4 ml of 20% TCA containing 5% TBA were added. The absorbance of the supernatant was recorded at 532 nm.

Nonspecific turbidity was corrected by subtracting the absorbance of the same at 600 nm. The content of TBARS was calculated using the extinction coefficient (155mM^{-1} cm^{-1}).

2.3. Histochemical staining method

For assay of the level of generation of $O_2^{\bullet -}$,histochemical staining method was used using nitro blue tetrazolium chloride (NBT) respectively, to stain the leaves by adopting the method of Wang et al. (2011) with slight modification.

The samples (3 leaves) from each treatment were immersed into 1 mg ml^{-1} NBT solution prepared in 10 mM phosphate buffer (pH 7.8) at ambient temperature under light for 6 hour. Blue (NBT staining) spots appeared; the stained samples were cleared in concentrated ethanol and kept in 70% ethanol and then pictures were taken with a NIKON digital camera (COOLPIX110).

2.4. Nitrogen assimilation

2.4.1. Determination of NR activity and N content

Leaf nitrate reductase (NR; EC1.7.99.4) activity was measured in fresh leaves by using the method of Kuo et al. (1982) by preparing the enzyme extract.

Leaf tissues (100 mg) were frozen in liquid N_2 and ground to a powder with mortar and pestle and then stored at −80°C. At 4°C, the powder was thawed for 10 min and homogenized in 250mM Tris-HCl buffer (pH 8.5), containing 10mM cysteine, 20mM FAD, 1.0mM EDTA, 1.0mM DTT and 10% (v/v) glycerol. At 4°C, the homogenate was centrifuged at 10,000 x *g* for 30min. NR activity was measured by adopting the method of Nakagawa et al. (1984) spectrophotometrically as the rate of nitrite production at 28°C. The assay mixture contained KNO_3 (10mM), HEPES (0.065M pH 7.0), NADH (0.5mM) in phosphate buffer (0.04mM, pH 7.2) and the enzyme extract in a final volume of 1.5ml. The reaction was started by the addition of NADH. After 15min of incubation, the reaction was terminated by the addition of 1.0 ml of 1.0 N HCl solution containing 1% sulphanilamide followed by the addition of 1.0 ml of 0.02% aqueous N-1-napthylethylene-di-amine di hydrochloride. The absorbance was read at 540 nm after 10 min.

Leaves N content was estimated by the Kjeldahl digestion method described by Lindner (1944). A 10 ml aliquot of the digested material was taken in a 50ml volumetric flask. To this, 2 ml of 2.5 N NaOH and 1.0 ml of 10% Na_2SiO_3 solutions were added to neutralize the excess of acid and to prevent turbidity, respectively. The volume was made up to the mark with de-ionized water. In a 10 ml graduated test tube, 5ml aliquot of this solution was taken and 0.5ml Nessler's reagent was added.

The contents of the test tubes were allowed to stand for 5 min for maximum color development. The optical density of the solution was read on a spectrophotometer at 525 nm.

2.5. Sulfur-assimilation

2.5.1. Assay of ATP-S and S content

Activity of ATP-sulphurylase activity(ATP-S; EC2.7.7.4) was assayed using molybdate-dependent formation of pyrophosphate as adopting the method of Lappartient and Touraine (1996).

Fresh leaf tissue (1.0 g) was ground at 4°C in a buffer consisting of 10mM Na_2EDTA, 20mMTris–HCl (pH 8.0), 2mM dithiothreitol (DTT) and 0.01g ml^{-1} PVP, using a 1:4 (w/v) tissue to buffer ratio. At 4°C, the homogenate material was centrifuged at 20,000 x *g* for 10 min. The supernatant was used for an in vitro ATP-S assay. In an Eppendorf tube, the reaction was initiated by adding 0.1ml of extract to 0.5ml of the reaction mixture, which contained 7mM $MgCl_2$, 5mM of Na_2MoO_4, 2mM of Na_2ATP and 0.032 units ml^{-1} of sulfate-free inorganic pyrophosphate in 80mM Tris–HCl buffer (pH 8.0). Another aliquot from the same extract was added to the same reaction mixture except that Na_2MoO_4 was absent. Reactions were incubated at 37°C for 15 min, after which phosphate was determined on a spectrophotometer by reading absorbance at 660nm. ATP-sulphurylase activity was calculated from a standard curve obtained from known concentrations of PO_4^{3-}.

The content of S was determined using the turbidimetric method of Chesnin and Yien (1950). A 5 ml aliquot was used for turbidity development in 25ml volumetric flask. Turbidity in 5 ml aliquot was initiated by adding 2.5 ml gum acacia (0.25%) solution, 1.0g $BaCl_2$ sieved through 40–60mm mesh and the volume was made to 25ml with deionized water. The contents of 25 ml volumetric flask were thoroughly shaken till $BaCl_2$ wholly dissolved. The values were recorded at 415nm within 10 min after the turbidity development. A blank was run simultaneously after each set of determination. The amount of sulphate was calculated with the help of a calibration curve drawn afresh using a series of K_2SO_4solutions.

2.5.2. Determination of cysteine content

Cysteine content in leaves was determined spectrophotometrically, adopting the method of Gaitonde (1967).

Fresh leaf (500 mg) was homogenized in 5% (w/v) ice-cold perchloric acid. The suspension was centrifuged at 2800 x *g* for 1 h at 5°C, and the supernatant was filtered through Whatman No.1 paper. One ml of the filtered solution was treated with acid ninhydrin reagent. The extinction was

read at 580 nm, and the amount of Cys was calculated with reference to a calibration curve obtained under similar conditions for standard cysteine.

2.5.3. Reduced glutathione content and redox state

Reduced glutathione was determined following the method of Anderson (1985).Glutathione was assayed by an enzymic recycling procedure in which it was sequentially oxidized by 5'5'-dithiobis-2-nitrobenzoic acid (DTNB) and reduced by NADPH in the presence of GR. For specific assay of GSSG, the GSH was masked by derivatization with 2-vinylpyridine.

Fresh leaves (500mg) were homogenized in 2ml of 5% sulphosalicylic acid undercold conditions. The homogenate was centrifuged at10, 000x *g* for 10min. To 0.5ml of supernatant, 0.6mlof phosphate buffer (100mM, pH 7.0) and 40μl of5,5-dithiobis-2-nitrobenzoic acid (DTNB) were added.After 2 min the absorbance was read at 412 nm. Redoxstate was presented as the ratio of GSH to oxidizedglutathione (GSSG).

2.6. Measurement of antioxidant enzymes

Fresh leaf tissues were homogenized using chilled mortar and pestle with an extraction buffer containing 0.05% (v/v) Triton X-100 and 1% (w/v) polyvinyl pyrrolidone (PVP) in potassium-phosphate buffer (100 mM, pH 7.0). The homogenized material was centrifuged at 4°C at 15000 x *g* for 20 min. The supernatant was used for the detection of superoxide dismutase (SOD) catalase (CAT) and glutathione reductase (GR). For the assay of ascorbate peroxidase (APX), the extraction buffer was augmented with 2 mM ascorbate.

2.6.1. SOD

Activity of superoxide dismutase (SOD; EC1.15.1.1) was determined according to the methods of Beyer and Fridovich (1987) and Giannopolitis and Ries (1977) by monitoring the inhibition of photochemical reduction of nitro blue tetrazolium (NBT).

Five ml of the reaction mixture containing 5.0mM HEPES (pH 7.6), 0.1mM ethylene diamine tetra acetic acid (EDTA), 50mMNa_2CO_3 (pH 10.0), 13mM methionine, 0.025% (v/v) triton X-100, 63 μmol NBT, 1.3 μmol riboflavin and the enzyme extract was illuminated for 15 min (360 μmol m^{-2} s^{-1}). A control set of experiment was also illuminated for correcting the background absorbance. A unit of SOD was defined as the amount of enzyme that inhibited the NBT reduction by 50% at 560 nm.

2.6.2. CAT

Activity of catalase (CAT; EC1.16.1.6) was determined by the method of Aebi (1984) by monitoring the disappearance of H_2O_2 at 240 nm. The reaction mixture (1.5ml) contained 13.2mM H_2O_2 in 50mM phosphate buffer (pH 7.0) and 0.1ml of enzyme extract. A control set was also illuminated for correcting the background absorbance.

2.6.3. APX

Activity of ascorbate peroxidise (APX; EC1.11.1.11) was determined by the method of Nakano and Asada (1981) by recording the decrease in absorbance of ascorbate at 290 nm. The assay mixture (1 ml) contained phosphate buffer (50mM, pH 7.0), 0.1mM EDTA, 0.5mMascorbate, 0.1mM H_2O_2, and the enzyme extract. APX activity was calculated using the extinction coefficient 2.8mM^{-1} cm^{-1}. One unit of the enzyme is the amount necessary to decompose 1μmol of substrate per min at 25°C.

2.6.4. GR

Activity of glutathione reductase (GR; EC1.6.4.2) was determined by the method of Foyer and Halliwell (1976) by monitoring the GSH-dependent oxidation of nicotinamide adenine dinucleotide phosphate (NADPH) at 340 nm. Three ml of the assay mixture containing phosphate buffer (25mM, pH 7.8), 0.5mM GSSG, 0.2mM NADPH, and the enzyme extract were used for measurement. The activity of GR was calculated using extinction coefficient 6.2mM^{-1} cm^{-1}. One unit of enzyme is the amount necessary to decompose 1μmol of NADPH min^{-1} at 25°C.

2.7. Proline metabolism

2.7.1. Determination of proline content

Proline content in leaf was determined spectrophotometric ally by adopting the ninhydrin method of Bates et al. (1973).

Fresh leaf samples (250mg) were homogenized in 3.0ml of 3% sulphosalicylic acid. The homogenate was reacted with 1.0ml each of acid ninhydrin and glacial acetic acid for 1 h in a test tube placed in a water bath at 100°C. The mixture was extracted with toluene and absorbance was recorded at 520nm.

2.7.2. Determination of glutamyl kinase and proline oxidase activity

To determine the activity of glutamyl kinase (GK; EC 2.7.2.11) and proline oxidase (POX; EC 1.5.99.8) enzyme extract was prepared by homogenizing 500 mg leaf sample in 0.1M Tris-HCl buffer (pH 7.5) at 4°C. The homogenate was centrifuged at 30000 x *g* for 30 min and pellet was collected and used as extract for assay of GK and POX. For GK enzyme activity extract was stored at -20°C.

2.7.2.1. GK activity

Activity of GK was assayed by the method of Hayzer and Leisinger (1980) with slight modification. The frozen sample was suspended in 10ml of 0.1M Tris–HCl buffer containing 1mM 1,4-dithiothreitol (DTT) to rupture the cell and centrifuged at 30000 x *g* for 30min. The assay mixture contained 50mM L-glutamate, 10mM ATP, 20mM $MgCl_2$, 100mM hydroxylamine HCl and 50mM Tris–HCl (pH 7.0) with 200µl of desalted extract in a final volume of 500µl. The reaction was started by the addition of enzyme extract. After 30 min of incubation at 37°C, the reaction was stopped by the addition of 1.0ml $FeCl_3.3H_2O$ (2.5% w/v) and trichloroacetic acid (TCA) (6%, w/v)in 2.5M HCl. Protein was precipitated and removed by centrifugation at 12000xg(4°C) and absorbance was recorded at 540nm. Activity of glutamyl kinase was expressed in U mg^{-1} protein. One Unit of the enzyme activity is defined as µg ofglutamylhydroxamate min^{-1} mg^{-1} protein. Glutamylhydroxamate was used asstandard.

2.7.2.2. POX activity

Proline oxidase activity was determined adopting the method of Huang andCavalieri (1979) with slight modification. The pellet was mixed with 1ml Tricine,KOH buffer (pH 7.5) containing 6M sucrose. This extract was used for the enzymeassay. The assay mixture contained 1.2ml of 50 mMTris–HCl buffer (pH 8.5), 1.2ml of 5mM $MgCl_2$, 0.1ml of 0.5mM NADP, 0.1ml of 1mM KCN, 0.1ml of 1mMphenazinemethosulfate (PSM), 0.1ml of 0.06mM 2, 6 dichlorophenolindo phenols (DCPIP) and 0.1ml of 0.1M proline in a final volume of 3ml. Theincrease in absorbance was recorded at 600 nm at 25°C using proline to initiate thereaction. Proline oxidase activity was expressed in Umg^{-1} protein. One Unit of activity is defined as mM DCPIP reduced min^{-1} mg^{-1} protein.

2.8. Ethylene biosynthesis

2.8.1. ACS activity

The activity of 1-aminocyclopropane-1-carboxylic acid synthase (ACS; EC4.4.1.14) was measured by adopting the methods of Avni et al. (1994) and Woeste et al. (1999).

Leaf tissue (500mg) was ground in 100mM HEPES buffer (pH 8.0) containing 4mMdithiothreitol (DTT), 2.5mMpyridoxal phosphate and 25% polyvinylpyrrolidone (PVP). The homogenized preparation was centrifuged at 12,000 x *g* for 15 min. One ml of the supernatant was placed in a 30ml tube and 0.1ml of 5 mMAdoMet was added and incubated for 2 h at 22°C. The ACC formed was determined by its conversion to ethylene by the addition of 0.1ml of 20 mM $HgCl_2$ followed by the addition of 0.1ml of a 1:1 mixture of saturated NaOH/NaCl and placed on ice for 10 min. for control set, AdoMet was not added.

2.8.2. Determination of ethylene biosynthesis

Ethylene evolution was measured by cutting 500mg leaf material into small pieces that were placed in 30ml tubes containing moist paper to minimize evaporation from the tissue and were stoppered with secure rubber caps and placed in light for 2 h under the same condition used for plant growth. Earlier experiment showed that 2 h incubation time was adequate for ethylene detection without the interference of wound-induced ethylene, which began after 2 h of leaf incubation. A 1ml gas sample from the tubes was withdrawn with a hypodermic syringe and assayed on a gas chromatograph (Nucon 5700, New Delhi, India) equipped with a 1.8m Porapack N (80-100 mesh) column, a flame ionization detector and data station. Nitrogen was used as the carrier gas. The flow rates of nitrogen, hydrogen and oxygen were 30, 30 and 300ml min^{-1}, respectively. The detector was at 150°C. Ethylene identification was based on the retention time and quantified by comparison with the peaks from standard ethylene concentration.

2.9. Measurements of photosynthetic and growth characteristics

Gas exchange parameters [stomatal conductance (gs), intercellular CO_2 concentration (Ci) and net photosynthesis (Pn)] were measured in fully expanded upper most leaves of plants in each treatment using Infrared Gas Analyzer (CID-340, Photosynthesis system, Bio-Science, USA) between 11:00 and 12:00 at light saturating intensity on a sunny day(PAR; 720 μmol m^{-2} s^{-1}) and at 370±75 μmol mol^{-1} atmospheric CO_2 concentrations).

Chlorophyll content was measured with the help of SPAD chlorophyll meter (SPAD 502 DL PLUS, Spectrum Technologies, USA).

The maximal PSII photochemical efficiency (Fv/Fm) of the fully expanded second leaf from top of plant was determined with the help of chlorophyll fluorometer (OS-30p; OptiSciences, Inc.).

Plants were uprooted carefully from the pots, washed to remove dust. Leaf area was measured with a leaf area meter (LA 211, Systronics, New Delhi, India). Plant dry mass was recorded after drying the sample in a hot air oven at 80°C till constant weight.

2.10. Electron microscopy

2.10.1. Scanning electron microscopy

Scanning electron microscopy (SEM) of leaf samples was carried out by adopting the method of Daud et al. [53]. Fresh leaf samples were taken from the axillary positions (leaves with 1.5 × 1.5cm in size) and were preferably air-dried in desiccators. Consequently, leaf samples were first fixed with 2.5% glutaraldehyde and 2% paraformaldehyde in 0.1M phosphate buffer (pH 7.0) in equal quantity for

more than 4h and then washed three times with phosphate buffer for 15min at each step. The samples were post-fixed with osmium tetraoxide in potassium phosphate buffer (pH 7.0) for 1 h and were subsequently repeatedly washed three times with the same phosphate buffer for 15 min at each step. The specimens were dehydrated by a graded series of ethanol (50, 70, 80, 90, 95 and 100%) for about 15–20 min at each step and transferred to the mixture of alcohol and isoamyl acetate in an equal ratio for about 30 min. Subsequently, the samples were transferred to pure isoamyl acetate for 1 h. Furthermore, specimens were dehydrated in Carl Zeiss EVO (Germany) scanning electron microscope critical point dryer with liquid CO_2. Finally, the dehydrated specimens were coated with gold–palladium and observed under Carl Zeiss EVO scanning electron microscope at extra high tension or high voltage at 15 kV and magnification of 150× or 1000×. The stomata were observed under SEM at 150×and 1000×, and the stomatal frequency was determined by counting the number of stomata in the microscope field of view.

2.11. Statistical analysis

Data were analyzed statistically and standard error (SE) was calculated using analysis of variance (ANOVA) by SPSS (ver. 17.0 Inc., USA) for windows and presented as mean±SE (n = 4). The least significant difference (LSD) was calculated for the significant data at $P < 0.05$. Bars showing the same letter were not significantly different by LSD test at $P < 0.05$.
